# Publication Rates, Ethnic and Sex Disparities in UK and Ireland Surgical Research Prize Presentations: An Analysis of Data From the Moynihan and Patey Prizes From 2000 to 2020

**DOI:** 10.1007/s00268-021-06268-0

**Published:** 2021-08-12

**Authors:** Jaspreet K. Seehra, Christopher Lewis-Lloyd, Amanda Koh, Elena Theophilidou, Prita Daliya, Alfred Adiamah, Dileep N. Lobo

**Affiliations:** 1 Gastrointestinal Surgery, Nottingham Digestive Diseases Centre and National Institute for Health Research Nottingham Biomedical Research Centre, Nottingham University Hospitals NHS Trust and University of Nottingham, Queen’s Medical Centre, Nottingham, NG7 2UH UK; 2 MRC Versus Arthritis Centre for Musculoskeletal Ageing Research, School of Life Sciences, Queen’s Medical Centre, University of Nottingham, Nottingham, NG7 2UH UK

## Abstract

**Background:**

Presentation at academic conferences is an important marker of research productivity. However, not all accepted abstracts progress to full publication, and there is anecdotal evidence suggesting an imbalance in sex and ethnicity amongst presenters. There is a lack of data evaluating the outcome of prize presentation sessions at academic surgical conferences in the UK. This study aimed to analyse the outcomes and demographics from presentations at prize sessions at two prestigious UK surgical conferences.

**Methods:**

This retrospective observational study compared data on all Moynihan (Association of Surgeons of Great Britain and Ireland) and Patey (Surgical Research Society) prize presentations from 2000 to 2020. The primary outcome was rate of publication. Secondary outcomes included demographic differences in sex and ethnicity, publication according to prize outcome, academic affiliation, time to publication, and journal impact factor.

**Results:**

Some 442 accepted abstracts were identified over the 21-year period, with 71.0% from the Moynihan sessions and 79.3% from the Patey sessions leading to full publications, with a median time to publication of 448 days (IQR 179–859) in journals with relatively high impact factors (median 5.00; IQR 3.15–6.36). Of the 442 prize presenters, 85 (19.2%) were female. The majority of the presenters were White males (211, 47.7%), followed by Asian males (112, 25.3%). However, there was a continuously increasing overall trend of female presenters from 2000 to 2020 (*P* = 0.019).

**Conclusion:**

Publication rates from the two prize sessions were high, with presenters publishing in journals with high impact factors. There, however, was a disparity in sex and ethnicity amongst presenters.

## Introduction

Academic surgical conferences provide a forum to showcase up-to-date surgical research through education, discussion and presentation of new work. Participation at conferences is an important marker of academic productivity. Although abstracts submitted to conferences are reviewed by a panel of scientific experts prior to acceptance, they are not subject to the same rigorous peer-review process of a journal publication. Whilst desirable, full publication is not always achievable and studies have reported that less than 50% of abstracts presented at surgical conferences progress to a full publication [1–4].

There is anecdotal evidence to suggest that imbalances in sex and ethnicity exist amongst presenters at conferences [5–8], which may reflect a lack of diversity within academic surgery. The lower female representation at academic conferences, in addition to the ongoing perceived challenges of balancing a demanding career and family life, further detracts female trainees from considering a career in academic surgery [5, 6]. Previous studies from the USA have highlighted that ethnic groups such as Hispanic, Asian and Afro-Caribbean doctors are under-represented in academia [7, 8]. However, similar data from the UK and Europe are sparse [9].

The Association of Surgeons of Great Britain and Ireland (ASGBI, https://www.asgbi.org.uk) annual congress and the Surgical Research Society (SRS, http://surgicalresearch.org.uk) annual meeting are two of the most prestigious academic surgical conferences in the UK and Republic of Ireland. The highest ranked abstracts submitted to these annual conferences are shortlisted for presentation at the Moynihan and Patey prize sessions, respectively, with one presenter being awarded each prize.

The aims of this retrospective observational study were to evaluate these two prestigious prize sessions in academic surgery in the UK and Republic of Ireland, assessing the publication rate of shortlisted presentations and prize winners, and identify whether there were any sex and ethnic disparities.

## Methods

This retrospective observational study collected data on all ASGBI Moynihan and SRS Patey prize presentations from 2000 to 2020. Data were collected by reviewing published abstracts of prize session presentations and contacting relevant persons within both organisations as well as liaising with library services of the Royal College of Surgeons of England. Data collected consisted of the first/presenting authors’ and senior authors’ sex, ethnicity and academic affiliation, conference prize presentation group, shortlisted presentation prize outcome, publication status and if published, time from presentation to publication and journal impact factor. Data on publication status were gathered by searching web browsers and relevant databases using whole text or keywords from abstract presentations to identify *PubMed*-cited original works of prize presentations. All data were freely available within the public domain, and therefore, ethical approval was not required.

### Primary and secondary outcomes

The primary outcome measure was the rate of conversion of abstracts to full publications. Secondary outcome measures included the overall differences in demographics of prize presenters and senior authors by sex and ethnicity, and temporal differences in demographics by prize, presentation prize outcome, academic affiliation, progression to article publication and if published, time to publication and journal impact factor.

### Variable definitions

Sex [10] and ethnicity of the first/presenting authors and senior authors were derived from their forename and surname listed on the published presentation abstract using “*Gender* (https://gender-api.com/) and *NamSor Version 2 diasporaBatch* (https://www.namsor.com/)” application programming interfaces (API), respectively. Ethnicity was coded into four main ethnic groups (White, Asian, Black, Other/Arab) as defined by the *Office for National Statistics* (https://www.ons.gov.uk/methodology/classificationsandstandards/measuringequality/ethnicgroupnationalidentityandreligion). The sex and ethnicity APIs are automated linguistic matching software programmes used routinely to identify sex and ethnicity and limit observer bias or discrimination [11, 12]. Throughout the study, sex is preferentially used, as biologically defined, whereas gender is a social construct encompassing individual gender identity.

Shortlisted presentations were assigned as nominees or winners depending on the outcome at each conference. Academic affiliation was categorised according to whether the presenter/first author was linked to a university and if so, whether the university was considered prestigious. Prestigious universities were classed as either *Russell Group* (https://russellgroup.ac.uk/) or *Ivy League* (https://ivyleague.com/) universities. Outside the UK and USA, according to the *Times Higher Education World University Rankings 2021* (https://www.timeshighereducation.com/), the top three universities within the Republic of Ireland *(Trinity College Dublin, Royal College of Surgeons in Ireland and University College Dublin)* and international universities ranked within the top 100 institutions worldwide were also considered prestigious. Presentations were categorised as published if a *PubMed* citable original article was identified in addition to the published abstract presentation that was authored by the presenter/first author and contained data presented at the conference. Time to publication was calculated in days from the date of conference presentation to the date of first online publication. Where article publications occurred prior to conference presentation the time to publication was recorded as 0 days. Journal impact factors were derived from the 2019 *Journal Citation Reports* published by *Clarivate* in 2020 (https://clarivate.com/webofsciencegroup/solutions/journal-citation-reports/). The journals where these studies were published were subclassified into surgical, medical, basic science/translational and miscellaneous based on their attributions on Scimago Journal and country rank (SJR).

### Statistical analysis

Data normality was assessed by visualising distribution plots, and descriptive statistics were used to report demographics including percentage frequencies, medians and interquartile ranges (IQR). Fisher’s exact, Chi-square (χ^2^) and Mann–Whitney U tests were used to compare categorical and continuous variables as appropriate. Univariate regression analysis with the likelihood ratio test was used to compare time trends by year and presented using 3-year rolling averages. Comparisons were made between prizes (Moynihan and Patey), winners and nominees, and by sex and ethnicity. All analyses were conducted using Stata Statistical Software v16.1 (StataCorp, College Station, Texas, USA) with significance set at the 95% level and *P* < 0.05 considered significant.

## Results

### Demographics of presenters

**Fig. 1:**
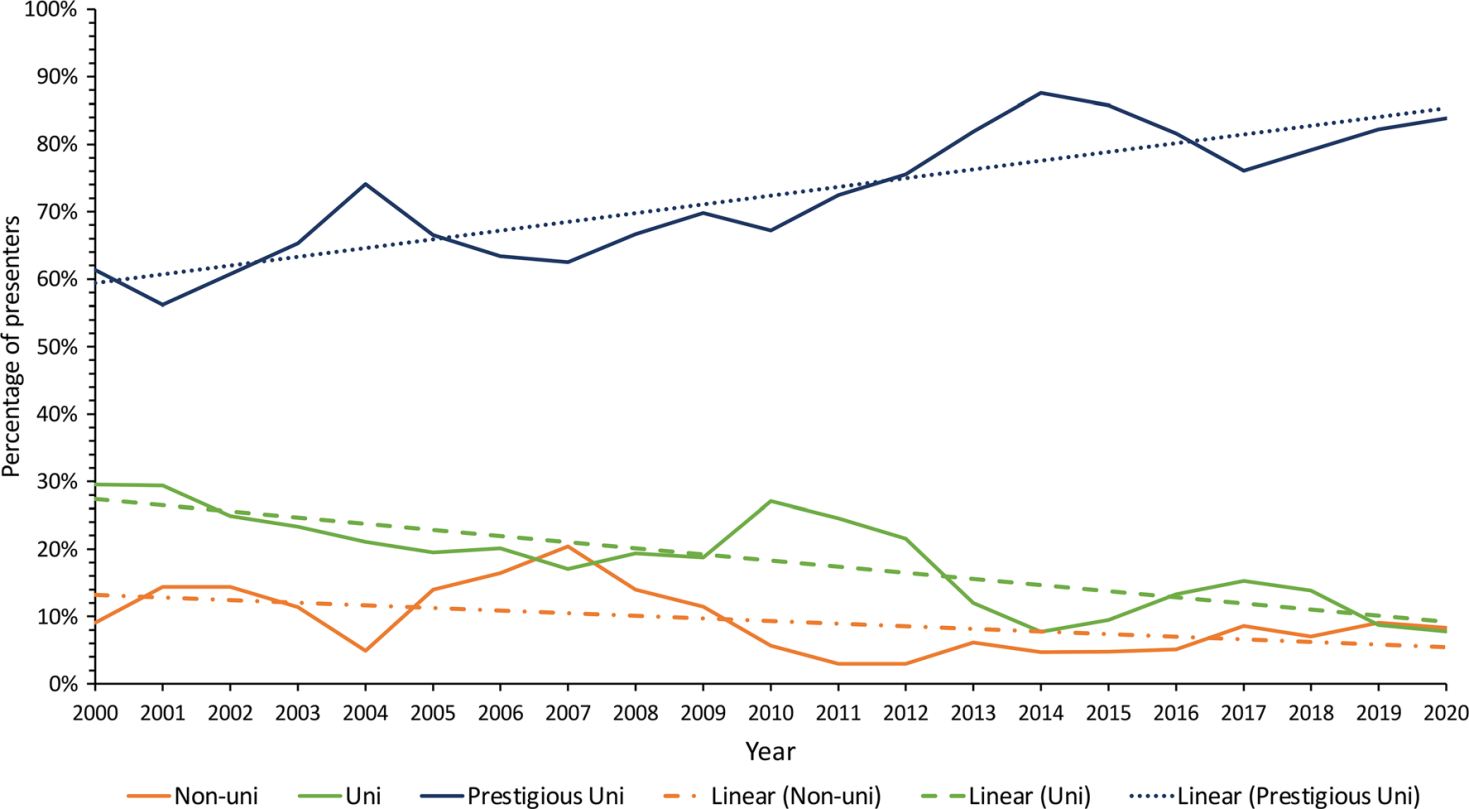
Proportion of Moynihan and Patey Prize presenters by university affiliation over time using a 3-year rolling average

The study identified 442 eligible presenters from 2000 to 2020, including 138 (31.2%) at the ASGBI Moynihan and 304 (68.8%) at the SRS Patey prize sessions. The general characteristics of the presenters are summarised in Table 1, with male presenters forming the majority (80.8% male *vs*. 19.2% female). Amongst the presenters, 61.1% were White, followed by 29.0% Asian, 0.5% Black, and 9.5% Other/Arab. Of the 442 presenters, 316 (71.5%) had affiliations with a prestigious university, 83 (18.8%) with a non-prestigious university, and 43 (9.7%) did not specify a university affiliation. Throughout 2000 to 2020, there was an increasing proportion of presenters having an affiliation to a prestigious university (*P* = 0.002, Fig. 1).

**Table 1:** Demographics of Moynihan and Patey prize presenters from 2000 to 2020 by sex

	Male	Female	*P*-value^d^	Total (*n* = 442)
	*n* = 357 (80.77%)	*n* = 85 (19.23%)		
	*n* (%)	*n* (%)		*n* (%)^g^
Prize				
Moynihan	115 (83.3%)	23 (16.7%)	0.357	138 (31.2%)
Patey	242 (79.6%)	62 (20.4%)		304 (68.8%)
Winner				
No	321 (80.3%)	79 (19.7%)	0.393	400 (90.5%)
Yes	36 (85.7%)	6 (14.3%)		42 (9.5%)
Ethnicity				
White	211 (78.2%)	59 (21.9%)	0.067^e^	270 (61.1%)
Asian	112 (87.5%)	16 (12.5%)		128 (29.0%)
Black	1 (50.0%)	1 (50.0%)		2 (0.5%)
Other/Arab	33 (78.6%)	9 (21.4%)		42 (9.5%)
Prestigious university^a^				
No	70 (83.3%)	14 (16.7%)	0.467	84 (19.0%)
Yes	250 (79.4%)	65 (20.6%)		315 (71.3%)
Non-university linked	37 (86.1%)	6 (13.9%)		43 (9.7%)
Abstract type				
Laboratory	253 (81.9%)	56 (18.1%)	0.368	309 (69.9%)
Clinical	104 (78.2%)	29 (21.8%)		133 (30.1%)
Published				
No	78 (75.73%)	25 (24.27%)	0.139	103 (23.3%)
Yes	279 (82.30%)	60 (17.70%)		339 (76.7%)
Journal type^b^				
Surgery	111 (84.7%)	20 (15.3%)	0.738	131 (38.6%)
Medicine	64 (81.0%)	15 (19.0%)		79 (23.3%)
Basic/translational	92 (81.4%)	21 (18.6%)		113 (33.3%)
Miscellaneous	12 (75.0%)	4 (25.0%)		16 (4.7%)
Time to publish (days)^c^				
Median (IQR)	427 (177–854)	528.5 (222.5–873)	0.538^f^	448 (179–859)
Journal impact factor^b^				
Median (IQR)	5.142 (3.184–6.785)	4.546 (2.74–5.676)	0.012^f^	4.997 (3.15–6.36)

Frequencies expressed as percentage row unless otherwise stated

IQR, Interquartile range

^a^Prestigious university includes Russell Group, Ivy League Universities and other Universities considered prestigious (Ireland: Trinity College, Royal College of Surgeons in Ireland and University College Dublin) (Netherlands: University of Amsterdam) (Canada: University of Toronto)

^b^Journal type *n* = 339, journal impact factor *n* = 338

^c^Articles published prior to conference presentation classed as 0 days. Male *n* = 279, Female *n* = 60, Overall *n* = 339

^d^Chi-square test

^e^Fisher's exact test

^f^Mann–Whitney *U* test

^g^Frequencies expressed as percentage column

### Progression to article publication

The majority of the research output presented at both Moynihan and Patey prize sessions led to publications (339 out of 442; 76.7%), with 71.0% from the Moynihan and 79.3% from the Patey prize sessions resulting in full-text publications (*P* = 0.057, Table 2). The median time between presentation and full-text publication was 448 days (IQR 179–859 days). There was no significant difference between the Moynihan and Patey prize groups (*P* = 0.468). The 98 full-text publications generated from the Moynihan prize group were published in journals with a median impact factor of 5.33 (IQR 3.36–5.79), whereas the 241 publications from the Patey prize group were accepted in journals with a median impact factor of 4.55 (IQR 3.15–6.60; *P* = 0.513).

**Table 2:** Demographics of Moynihan and Patey prize presenters from 2000 to 2020 by prize

	Moynihan prize	Patey prize	*P*-value^d^
	*n* = 138 (31.2%)	*n* = 304 (68.8%)	
	*n* (%)	*n* (%)	
Winner			
No	117 (84.8%)	283 (93.1%)	0.006
Yes	21 (15.2%)	21 (6.9%)	
Sex			
Male	115 (83.3%)	242 (79.6%)	0.357
Female	23 (16.7%)	62 (20.4%)	
Ethnicity			
White	89 (64.5%)	181 (59.5%)	0.695^e^
Asian	38 (27.5%)	90 (29.6%)	
Black	0 (0%)	2 (0.7%)	
Other/Arab	11 (8.0%)	31 (10.2%)	
Prestigious university^a^			
No	31 (22.5%)	53 (17.4%)	0.008
Yes	86 (62.3%)	229 (75.3%)	
Non-university linked	21 (15.2%)	22 (7.2%)	
Abstract Type			
Laboratory	66 (47.8%)	243 (79.9%)	< 0.0001
Clinical	72 (52.2%)	61 (20.1%)	
Published			
No	40 (29.0%)	63 (20.7%)	0.057
Yes	98 (71.0%)	241 (79.3%)	
Journal type^b^			
Surgery	48 (49.0%)	83 (34.4%)	0.017
Medicine	23 (23.5%)	56 (23.2%)	
Basic/translational	21 (21.4%)	92 (38.2%)	
Miscellaneous	6 (6.1%)	10 (4.2%)	
Time to publish (days)^c^			
Median (IQR)	539.5 (223–842)	420 (177–859)	0.468^f^
Journal impact factor^b^			
Median (IQR)	5.328 (3.357–5.791)	4.546 (3.149–6.604)	0.513^f^

Frequencies expressed as percentage column unless otherwise stated

IQR, Interquartile range

^a^Prestigious university includes Russell Group, Ivy League Universities and other Universities considered prestigious (Ireland: Trinity College, Royal College of Surgeons in Ireland and University College Dublin) (Netherlands: University of Amsterdam) (Canada: University of Toronto)

^b^Journal type *n* = 339, journal impact factor *n* = 338

^c^Articles published prior to conference presentation classed as 0 days. Male *n* = 279, Female *n* = 60, Overall *n* = 339

^d^Chi-square test

^e^Fisher's exact test

^f^Mann–Whitney *U* test

Overall, the majority of presentations were on laboratory-based/basic science research (69.9%) and 30.1% were on clinical research. Between the two prize sessions, presentations from the Patey prize sessions were more likely to be laboratory-based, 79.9% vs 47.8% (*p* < 0.001). The journals where these studies were published were surgical (38.6%), medical (23.3%), basic science/translational (33.3%) and miscellaneous (4.7%), with differences found in the journal type between the Moynihan and Patey prize sessions (*P* = 0.017), which mirrors the types of research presented (Table 2).

### Presentation prize outcome

**Fig. 2:**
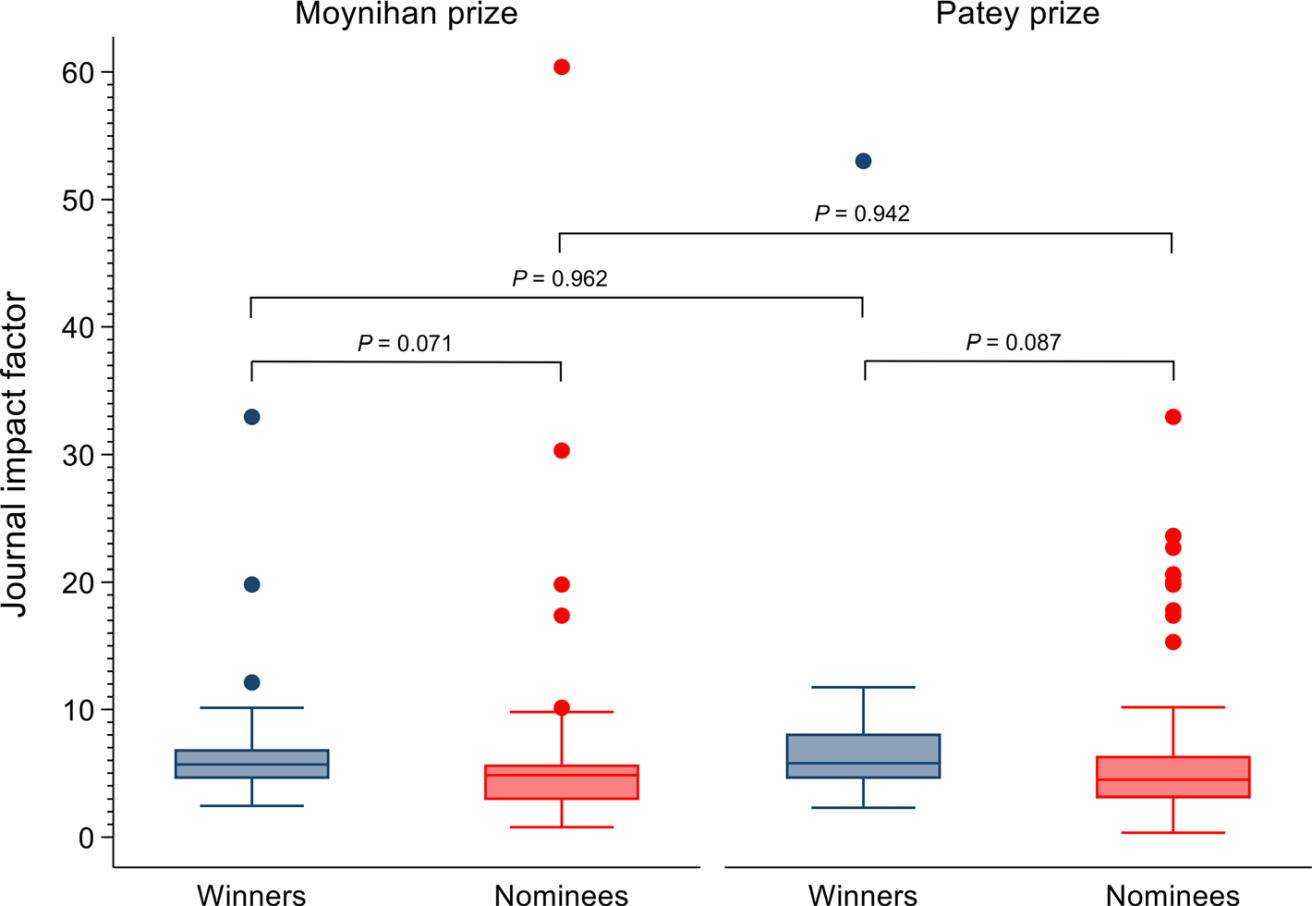
Box plots of journal impact factor for nominees and winners of the Moynihan and Patey prizes. Solid lines represent medians, boxes interquartile ranges, whiskers ranges and dots outliers. Mann–Whitney U test used to derive *P* values

Detailed information on the characteristics for both prize nominees and winners is presented in Table 3. Prize winners were more likely to publish their research output compared with their prize nominee counterparts (85.7% and 75.8%, respectively), but this was not statistically significant (*P* = 0.147). Prize winners were more likely to publish in journals with a higher impact factor (winner = 5.68, IQR 4.55–7.57; nominee = 4.57, IQR 3.03–6.03; *P* = 0.012) (Fig. 2). There were no statistically significant differences in the sex, ethnicity, university affiliation, and time to publication between the two prize groups (Table 3).

**Table 3:** Demographics of Moynihan and Patey prize presenters from 2000 to 2020 by prize winners

	Nominee	Winner	*P*-value^d^
	*n* = 400 (90.5%)	*n* = 42 (9.5%)	
	*n* (%)	*n* (%)	
Prize^g^			
Moynihan	117 (84.8%)	21 (15.2%)	0.006
Patey	283 (93.1%)	21 (6.9%)	
Sex			
Male	321 (80.2%)	36 (85.7%)	0.393
Female	79 (19.8%)	6 (14.3%)	
Ethnicity			
White	242 (60.5%)	28 (66.7%)	0.674^e^
Asian	116 (29.0%)	12 (28.6%)	
Black	2 (0.5%)	0 (0.0%)	
Other/Arab	40 (10.0%)	2 (4.8%)	
Prestigious university^a^			
No	78 (19.5%)	6 (14.3%)	0.547
Yes	282 (70.5%)	33 (78.6%)	
Non-university linked	40 (10.0%)	3 (7.1%)	
Abstract Type			
Laboratory	274 (68.5%)	35 (83.3%)	0.046
Clinical	126 (31.5%)	7 (16.7%)	
Published			
No	97 (24.3%)	6 (14.3%)	0.147
Yes	303 (75.7%)	36 (85.7%)	
Journal type^b^			
Surgery	124 (40.9%)	7 (19.4%)	0.063
Medicine	70 (23.1%)	9 (25.0%)	
Basic/translational	95 (31.4%)	18 (50.0%)	
Miscellaneous	14 (4.6%)	2 (5.6%)	
Time to publish (days)^c^			
Median (IQR)	453 (193–859)	427 (145–889)	0.776^f^
Journal impact factor^b^			
Median (IQR)	4.568 (3.027–6.033)	5.676 (4.546–7.565)	0.012^f^

Frequencies expressed as percentage column unless otherwise stated

IQR, Interquartile range

^a^Prestigious university includes Russell Group, Ivy League Universities and other Universities considered prestigious (Ireland: Trinity College, Royal College of Surgeons in Ireland and University College Dublin) (Netherlands: University of Amsterdam) (Canada: University of Toronto)

^b^Journal type n = 339, journal impact factor *n* = 338

^c^Articles published prior to conference presentation classed as 0 days. Male *n* = 279, Female *n* = 60, Overall *n* = 339

^d^Chi-square test

^e^Fisher's exact test

^f^Mann–Whitney *U* test

^g^Frequencies expressed as percentage row

### Comparison of sex and ethnicity of prize presenter and senior author

There was a significant disparity in sex amongst the Moynihan and Patey prize presenters, with 80.8% of the overall prize presenters being male (Table 1). Between male and female presenters, there were no statistically significant differences in the type of conference, rate of winning, ethnicity, academic affiliation, and progression to article publication (Table 1). Out of the successful publications, male authors were more likely to publish in a journal with a higher impact factor (male = 5.14, IQR 3.18–6.79; female = 4.55, IQR 2.74–5.68; *P* = 0.020).

**Fig. 3:**
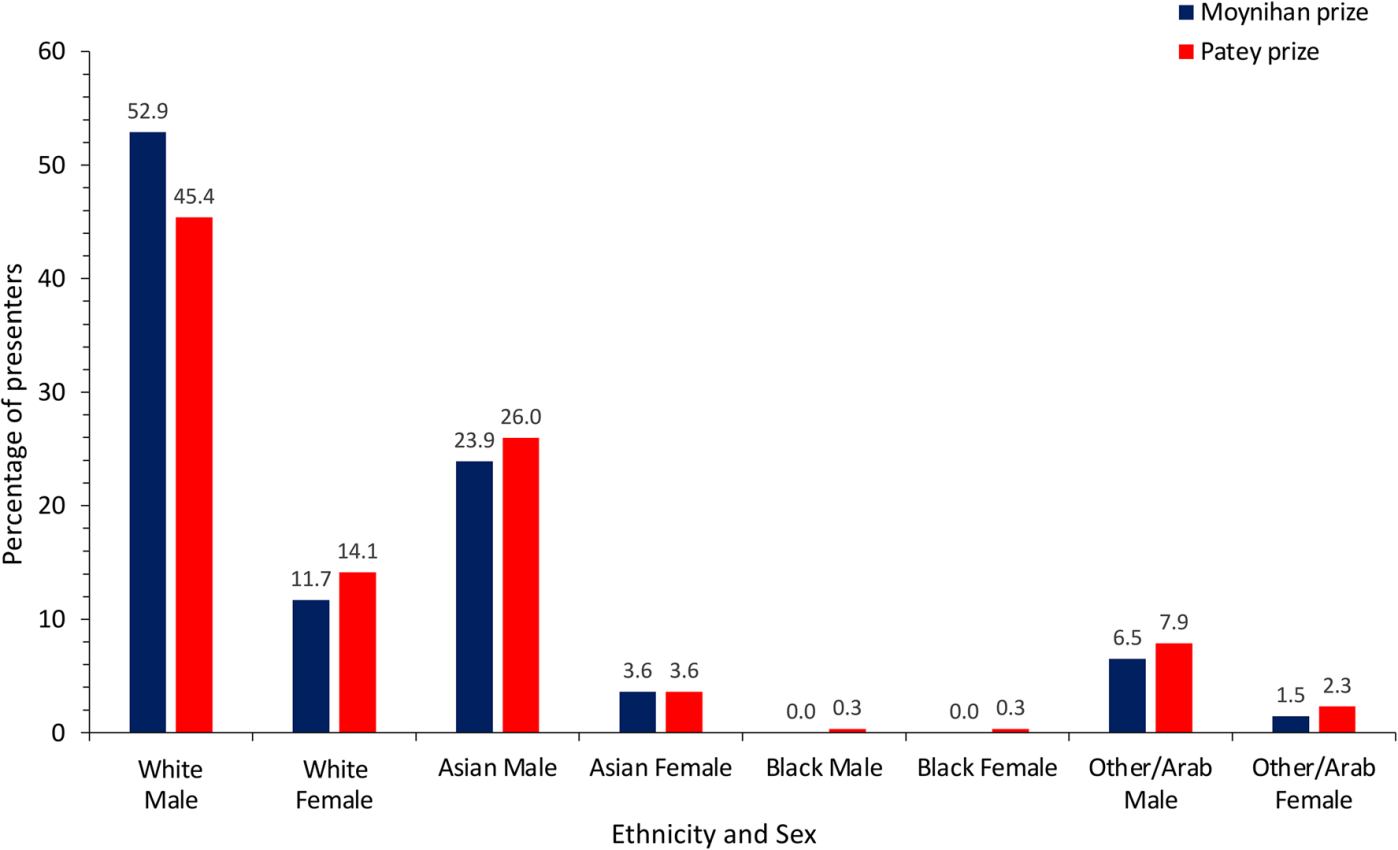
Proportions of Moynihan and Patey prize presenters from 2000 to 2020 by ethnicity and sex

**Fig. 4:**
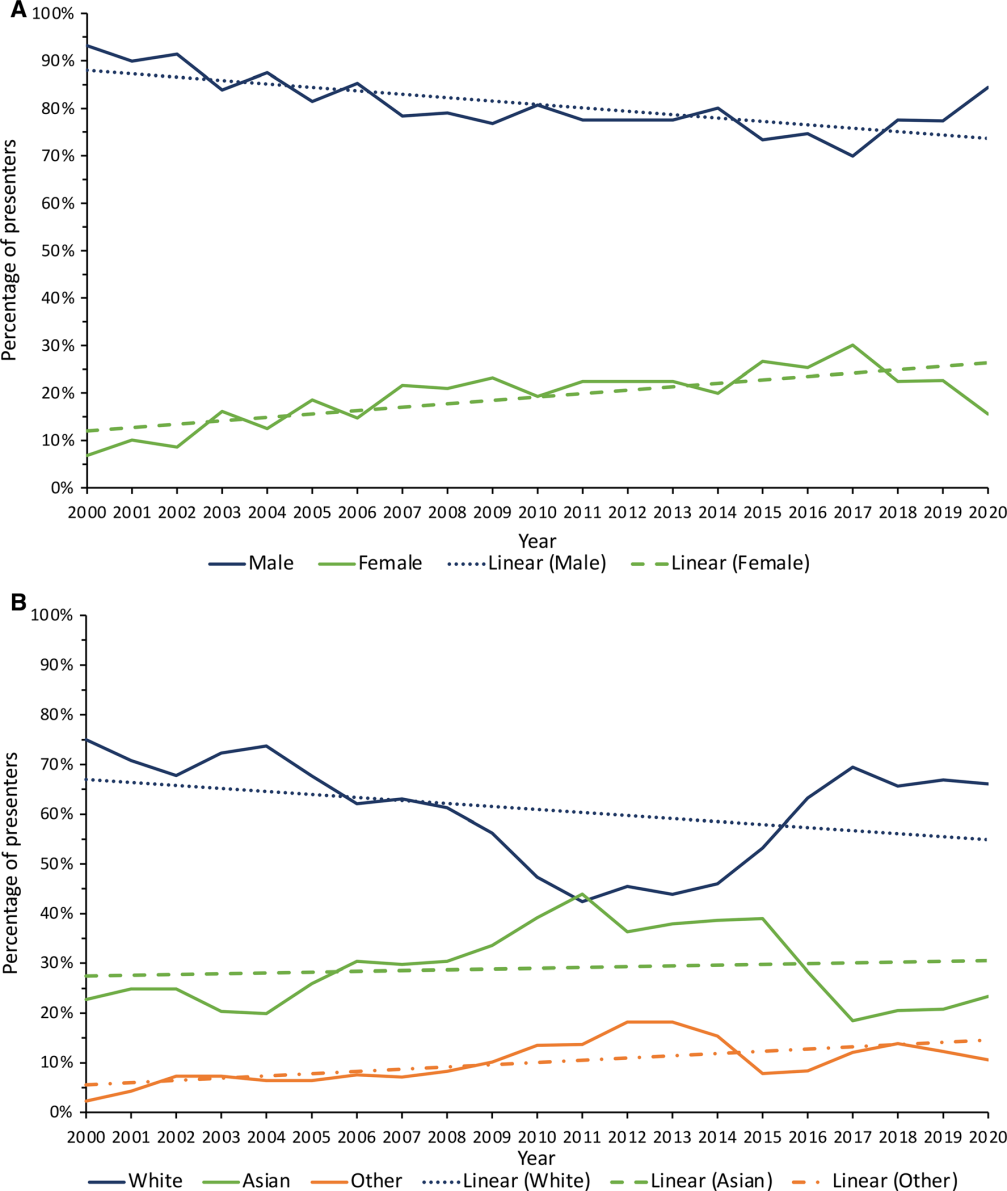
**a** Proportion of Moynihan and Patey prize presenters by sex over time using a 3-year rolling average. Chi-square (χ^2^) test for trend, *P* = 0.019. Likelihood ratio test trend, *P* = 0.019. **b** Proportion of Moynihan and Patey prize presenters by ethnicity over time using a 3-year rolling average. Chi-square (χ^2^) test for trend, *P* = 0.147. Likelihood ratio test trend, *P* = 0.058

Amongst presenters at both conferences, nearly half (211, 47.8%) were White men, followed by Asian men (112, 25.3%), as shown in Fig. 3. There were fewer female presenters in each ethnicity group, with the exception of the Black group, where there were equally low numbers (0.23% each). Despite this, there was a continuously increasing trend of overall female presenters from 2000 to 2020 (Fig. 4a), which was statistically significant (likelihood ratio test trend, *P* = 0.019). Throughout the 21-year period, there was a steadily increasing trend of non-White presenters across both conferences (*P* = 0.002, Fig. 4b).

**Fig. 5:**
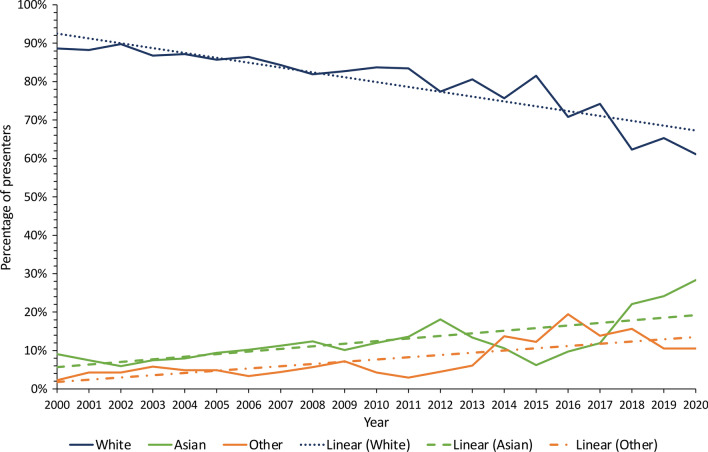
Proportion of Moynihan and Patey prize senior authors by ethnicity over time using a 3-year rolling average. Chi-square (χ^2^) test for trend, *P* = 0.112. Likelihood ratio test trend, *P* = 0.019

Of the abstracts presented at both prize sessions, an overwhelming majority of the senior authors were male and White (88.0%) with a significant disparity noted in female senior authors ((14.0%, *P* = 0.027) Table 4). Despite an increasing trend of non-White senior authors from 2000 to 2020 (Fig. 5), this was not statistically significant (*p* = 0.058).

**Table 4:** Demographics of Moynihan and Patey prize senior authors from 2000 to 2020 by sex and ethnicity

	Male *n* = 380 (86.0%)	Female *n* = 62 (14.0%)	*P*-value^d^
	*n* (%)	*n* (%)	
Prize			
Moynihan	124 (89.9%)	14 (10.1%)	0.114
Patey	256 (84.2%)	48 (15.8%)	
Winner			
No	342 (85.5%)	58 (14.5%)	0.378
Yes	38 (90.5%)	4 (9.5%)	
Ethnicity			
White	315 (88.0%)	43 (12.0%)	0.027
Asian	38 (74.5%)	13 (25.5%)	
Black	0 (0.0%)	0 (0.0%)	
Other/Arab	27 (81.8%)	6 (18.2%)	
Prestigious university^a^			
No	74 (88.1%)	10 (11.9%)	0.475
Yes	267 (84.8%)	48 (15.2%)	
Non-university linked	39 (90.7%)	4 (9.3%)	
Abstract Type			
Laboratory	265 (85.8%)	44 (14.2%)	0.845
Clinical	115 (86.5%)	18 (13.5%)	
Published			
No	88 (85.4%)	15 (14.6%)	0.858
Yes	292 (86.1%)	47 (13.9%)	
Journal type^b^			
Surgery	113 (86.3%)	18 (13.7%)	0.379
Medicine	71 (89.9%)	8 (10.1%)	
Basic/translational	93 (82.3%)	20 (17.7%)	
Miscellaneous	15 (93.8%)	1 (6.2%)	
Time to publish (days)^c^			
Median (IQR)	432 (189.5–821.5)	577 (148–1090)	0.241^e^
Journal impact factor^b^			
Median (IQR)	5.028 (3.177–6.36)	4.366 (2.557–5.791)	0.360^e^

Frequencies expressed as percentage row unless otherwise stated

IQR, Interquartile range

^a^Prestigious university includes Russell Group, Ivy League Universities and other Universities considered prestigious (Ireland: Trinity College, Royal College of Surgeons in Ireland and University College Dublin) (Netherlands: University of Amsterdam) (Canada: University of Toronto)

^b^Journal type *n* = 339, journal impact factor *n* = 338

^c^Articles published prior to conference presentation classed as 0 days. Male *n* = 279, Female *n* = 60, Overall *n* = 339

^d^Chi-square test

^e^Mann–Whitney *U* test

## Discussion

### Publication rates and their impact

This study found that the abstracts presented at the ASGBI Moynihan and SRS Patey prize sessions resulted in a high rate of publication following presentation, and with an acceptable time to publication. Nearly 71% of abstracts from the ASGBI Moynihan and 79% from the SRS Patey prize sessions led to publications. The median time from presentation to publication was 448 days, reflecting the length of the peer review and publication process [13]. Presenters at the two prize sessions were likely to publish in highly respected biomedical journals, with prize winners being more likely to publish their research output in journals with a higher impact factor. The Patey prize sessions had a higher proportion of basic science research compared with the Moynihan prize, and this influenced the types of journal the studies were published in subsequently.

### Sex and ethnic disparity

Interestingly, there was significant disparity in sex across both prize sessions, with male presenters forming the majority at 80%. This was consistent in both the Moynihan and Patey prize sessions. However, there was a steadily increasing trend of female presenters, rising from 15% in 2000 to 33% in 2020. Additionally, ethnicity subgroup analysis highlighted that the majority of presenters were White, followed by Asians. Across both prize sessions over the 21-year period, only one Black female, one Black male and 16 Asian female presenters were recorded out of a total of 442 presenters. The significant sex and ethnicity differences persisted even amongst senior authors. A focus should be placed on encouraging more women and ethnic minority groups to consider academic surgery as a potential career choice early on in their training. Additionally, educating medical students on study design, statistics and the range of possibilities that a career in academia can bring is paramount to fostering interest. Finally, embedding mentorship and flexibility in the different pathways into academic surgery might encourage future aspiring trainees to consider this as a career option.

### Current literature

A Cochrane review [2] of over 300,000 abstracts of studies presented at scientific meetings found that only 37% resulted in full publication, which is much lower than the 76.7% conversion rate found in the present study. The review [2] suggested that higher-quality presentations with a superior study design led to an increased likelihood of full publication. In the present analysis, the majority of studies (70%) comprised basic science research, and this may therefore explain the higher publication rate.

Academic surgery is historically perceived to be one of the more difficult career options for women [14, 15]. A recent study by the Royal College of Surgeons of England showed that only 13.2% [16] of consultant surgeons in 2020 were female, compared with 37.5% [17] in other specialties. This trend is consistent across training, with 34.8% of all surgical trainees being female compared with other specialties where over half (56.6%) were female [18]. The paucity of female presenters at academic conferences, as found in the present study, has been highlighted previously as an ongoing issue [5, 19, 20]. It is important to note that whilst the issue of representation was a factor, the quality of work once shortlisted in terms of prize winners and publication rate did not differ between male and female presenters. It has been argued that women in surgery are less likely to pursue academic positions for various reasons including lack of mentoring, different career motivations, disproportionate childcare responsibilities, sexual harassment or other sex-based discrimination [21]. Thompson-Burdine et al. [15] highlighted the need to understand these complex issues for progress to be made through institutional policies and relational interactions. Women in Surgery [22] is a well-celebrated national initiative started 30 years ago, dedicated to encourage and inspire women to fulfil a successful career in surgery.

Although the issue of ethnic diversity in surgery has received more coverage in the USA [23, 24], there is a lack of data in the UK and Europe. Unlike the Women in Surgery initiative [22], there is no equivalent network for surgeons of colour. The first comprehensive diversity report [25] recently published by the Royal College of Surgeons of England identified the lack of diversity within the surgical profession as a whole and highlighted the barriers to progression faced by trainees from the ethnic minority groups. This ranged from overt bullying and racial discrimination, fear of judgement from colleagues, to microaggression. White doctors were more likely to be accepted into a surgical training programme when compared to their non-White counterparts (80.4% vs 70.5%) [25], and non-White surgical trainees experienced a higher rate of bullying (10.2% vs 6%) [25].

### Limitations

Analysis of the Moynihan and Patey prize sessions at the ASGBI and SRS conferences, respectively, may not be representative of the remaining sessions at the conferences. However, the two prestigious prize sessions are important in highlighting the best of surgical research. Additionally, prestigious prize sessions provide an opportunity to showcase the breadth and excellence of surgical research across the UK and Republic of Ireland. “Gender” API and ethnicity software programmes were used for the names of presenters and senior authors and could be subject to some erroneous findings. However, both systems have been validated in multiple studies [11, 12]. For all previous winners or shortlisted presenters known to the team, matching of sex and ethnicity were undertaken and compared with the results of both software packages, achieving 100% and 98% concordance on sex and ethnicity, respectively. Gender identity was not taken into account due to data not being widely available and the importance of ensuring anonymity. Additionally, although the Asian subcontinent is home to a large number of countries and there exists a large variation between the experiences amongst the different cultures, this current retrospective study did not undertake a subgroup analysis to differentiate between presenters from the East and South Asia due to small numbers. Finally, not all papers presented in the year 2020 were likely to have been published by the time of this analysis, given the median time to publication found here was 448 days**.**

### Conclusions

Prestigious prize sessions highlight important and impactful academic research in surgery. Associated publication rates were high, with prize winners publishing in journals with high impact factors. The significant disparity in sex and ethnicity of both presenters and senior authors, across both prize sessions, reflects the current state of academic surgery. More needs to be done, to address this imbalance and encourage diversity and representation across the echelons of academic surgery.

## References

[1] Rao AR, Beatty JD, Laniado M, Motiwala HG, Karim OM (2006). Publication rate of abstracts presented at the British Association of Urological Surgeons Annual Meeting. BJU Int.

[2] Scherer RW, Meerpohl JJ, Pfeifer N, Schmucker C, Schwarzer G, von Elm E (2018) Full publication of results initially presented in abstracts. Cochrane Database Syst Rev 11:MR000005 10.1002/14651858.MR000005.pub4 PMC7073270 30480762

[3] Dobson H, Wall S (2016). Publication rates of studies presented at the International Society of Craniofacial Surgery Congress. J Craniofac Surg.

[4] Khajehnoori M, Stupart D, Watters D (2018). Publication rate of General Surgery abstracts presented at the Royal Australasian College of Surgeons Annual Scientific Congress. ANZ J Surg.

[5] Gerull KM, Wahba BM, Goldin LM, McAllister J, Wright A, Cochran A (2020). Representation of women in speaking roles at surgical conferences. Am J Surg.

[6] Seemann NM, Webster F, Holden HA, Moulton CA, Baxter N, Desjardins C (2016). Women in academic surgery: why is the playing field still not level?. Am J Surg.

[7] Abelson JS, Symer MM, Yeo HL, Butler PD, Dolan PT, Moo TA (2018). Surgical time out: our counts are still short on racial diversity in academic surgery. Am J Surg.

[8] Butler PD, Longaker MT, Britt LD (2008). Major deficit in the number of underrepresented minority academic surgeons persists. Ann Surg.

[9] Joseph JP, Joseph AO, Jayanthi NVG, Pereira B, Gahir J (2020). BAME underrepresentation in surgery leadership in the UK and Ireland in 2020: an uncomfortable truth. Bull R Coll Surg Engl.

[10] Bewley S, McCartney M, Meads C, Rogers A (2021) Sex, gender, and medical data. BMJ 372:n735 10.1136/bmj.n735 33741563

[11] Khan MS, Lakha F, Tan MMJ, Singh SR, Quek RYC, Han E (2019). More talk than action: gender and ethnic diversity in leading public health universities. Lancet.

[12] Morgan R, Lundine J, Irwin B, Grepin KA (2019). Gendered geography: an analysis of authors in The Lancet Global Health. Lancet Glob Health.

[13] Björk B-C, Solomon D (2013). The publishing delay in scholarly peer-reviewed journals. Jo Informetr.

[14] Greenberg CC (2017) Association for Academic Surgery presidential address: sticky floors and glass ceilings. J Surg Res 219:ix-xviii 10.1016/j.jss.2017.09.006 29078918

[15] Thompson-Burdine JA, Telem DA, Waljee JF, Newman EA, Coleman DM, Stoll HI, et al. (2019) Defining barriers and facilitators to advancement for women in academic surgery. JAMA Netw Open 2:e1910228 10.1001/jamanetworkopen.2019.10228 PMC6724152 31469392

[16] The Royal College of Surgeons of England (2021) Statistics: women in surgery. https://www.rcseng.ac.uk/careers-in-surgery/women-in-surgery/statistics/ (accessed June 24 2021)

[17] NHS Digital (2020) NHS Workforce Statistics - March 2020. https://digital.nhs.uk/data-and-information/publications/statistical/nhs-workforce-statistics/march-2020 (accessed June 24 2021)

[18] Moberly T (2018) Number of women entering medical school rises after decade of decline. BMJ 360:k254

[19] Dumitra TC, Trepanier M, Lee L, Fried GM, Mueller CL, Jones DB (2020). Gender distribution of speakers on panels at the Society of American Gastrointestinal and Endoscopic Surgeons (SAGES) annual meeting. Surg Endosc.

[20] Wilcox AR, Trooboff SW, Lai CS, Turner PL, Wong SL (2019). Trends in gender representation at the American College of Surgeons Clinical Congress and the Academic Surgical Congress: a mixed picture of progress. J Am Coll Surg.

[21] Schroen AT, Brownstein MR, Sheldon GF (2004). Women in academic general surgery. Acad Med.

[22] The Royal College of Surgeons of England (2021) Women in surgery. https://www.rcseng.ac.uk/careers-in-surgery/women-in-surgery/ (accessed June 24 2021)

[23] Abelson JS, Wong NZ, Symer M, Eckenrode G, Watkins A, Yeo HL (2018). Racial and ethnic disparities in promotion and retention of academic surgeons. Am J Surg.

[24] Aggarwal A, Rosen CB, Nehemiah A, Maina I, Kelz RR, Aarons CB (2021). Is there color or sex behind the mask and sterile blue? Examining sex and racial demographics within academic surgery. Ann Surg.

[25] The Royal College of Surgeons of England (2021) The Royal College – Our Professional Home. An independent review on diversity and inclusion for the Royal College of Surgeons of England: An exciting call for radical change. The Royal College of Surgeons of England, London. https://www.rcseng.ac.uk/-/media/files/rcs/about-rcs/about-our-mission/rcs-diversity-report-30-march-1.pdf. Accessed June 21 2021

